# Double-Edged Effects of Social Strategies on the Well-Being of Autistic People: Impact of Self-Perceived Effort and Efficacy

**DOI:** 10.3390/brainsci14100962

**Published:** 2024-09-25

**Authors:** Ren Funawatari, Motofumi Sumiya, Toshiki Iwabuchi, Atsushi Senju

**Affiliations:** 1 United Graduate School of Child Development, Osaka University, Kanazawa University, Hamamatsu University School of Medicine, Chiba University, and University of Fukui, Osaka 565-0871, Japan; renfuna@hama-med.ac.jp (R.F.); sumiya@hama-med.ac.jp (M.S.); iwabuchi@hama-med.ac.jp (T.I.); 2 Research Center for Child Mental Development, Hamamatsu University School of Medicine, Hamamatsu 431-3192, Japan

**Keywords:** autism, social strategy, camouflaging, masking, well-being

## Abstract

Background/Objectives: Autistic people employ various social strategies to form and maintain interpersonal relationships in their daily environments. These strategies can help autistic people with social interactions (leading to self-perceived efficacy of using social strategies), but can also lead to cognitive fatigue (self-perceived effort of using social strategies). However, previous studies have focused primarily on self-perceived effort, overlooking the self-perceived efficacy of using social strategies, and the balance between self-perceived effort and efficacy. To address this gap, this study examined the impact of autistic people’s use of social strategies on their well-being, focusing on self-perceived effort, self-perceived efficacy, and their interaction effect. Methods: An online survey was conducted among self-reported autistic people in Japan aged 18–65 years, using a modified Compensation Checklist. Data from 104 self-reported autistic participants were analyzed using linear regression. Results: High self-perceived effort in using social strategies was negatively associated with well-being, whereas high self-perceived efficacy was positively associated with well-being. The interaction effect between effort and efficacy was not significant. These results were supported even when loneliness was used as an index of social well-being. Additionally, the number of strategies used by an autistic person was positively associated with well-being. Conclusions: This study highlights the double-edged effect of autistic people using social strategies, and that using a broader repertoire of social strategies may improve the well-being of autistic people. These findings call for a nuanced approach by researchers and clinicians considering both the positive and negative aspects of using social strategies.

## 1. Introduction

Autism is a neurodevelopmental condition that manifests early in life, leading to challenges in social communication and interaction, alongside a tendency toward restricted and repetitive interests and behaviors. These traits can make it difficult for autistic people to form and maintain interpersonal relationships in daily environments such as schools and workplaces [1]. To navigate these social landscapes, many autistic people employ various strategies, which may include hiding their autistic traits and developing alternative cognitive and behavioral approaches to compensate for their social difficulties [2]. These strategies could affect their well-being in both positive and negative ways. While they help autistic people navigate social interactions in daily life, they are also associated with cognitive fatigue [3,4]. Understanding how these strategies influence autistic people’s well-being is crucial for improving their quality of life.

Previous research has primarily focused on their potential negative impacts on autistic people, raising concerns about their overall effect on well-being. For example, when autistic people use social strategies, it may make them feel overlooked, under supported, burnt out, and inauthentic [3,4]; it may also develop a sense of low self-esteem and identity confusion [4], as well as exhaustion due to high cognitive demands [3,5], thus leading to loneliness and anxiety [6]. Most notably, the majority of studies that adopted the Camouflaging Autistic Traits Questionnaire (CAT-Q), the first standardized self-report questionnaire to assess the use of social strategies [7], reported negative effects of these strategies on well-being, such as increasing depression, anxiety, and stress (see [8,9] for reviews). These studies have indicated that using social strategies with high cognitive demand is linked to poor mental health in autistic people [3]. In other words, using social strategies with high self-perceived *effort* (the effort required to use social strategies or how tiring it is to use them) may lead to an overwhelming cognitive load and psychological distress, resulting in a negative impact on their well-being.

However, by focusing primarily on the negative consequences of using social strategies, the line of research we highlighted above possibly took our focus away from other lines of evidence, which indicated potential benefits of the use of social strategies for autistic people’s well-being. For example, qualitative accounts from autistic people reveal the potential benefits of social strategies. These social strategies can help autistic people navigate social interactions and live in society [3,4,10] by offering a practical way for some autistic people to achieve and function in a largely non-autistic society [4], managing others’ impressions about them [4,10], and acting as a defense mechanism from stressful social encounters [3]. In addition, for some autistic people, the use of social strategies can become naturalized and automatic, without them losing their sense of true self [5]. When used efficiently, social strategies can provide tangible benefits, such as facilitating social participation and improving daily functioning. The self-perceived *efficacy* of these strategies—how well autistic people believe these strategies help them live in society—can enhance their well-being by offering them practical benefits.

Furthermore, a balance between self-perceived effort and efficacy of the social strategies used may also influence well-being, as suggested by the previous literature in the field of coping with non-autistic populations [11,12]. For example, when self-perceived effort is low but self-perceived efficacy is high, a reduced risk of heart disease [13] and depression [14] has been observed. A similar effect of balance between effort and efficacy can influence the impact of social strategies on the well-being of autistic people.

Therefore, to understand how social strategies affect the well-being of autistic people more comprehensively, it is essential to consider self-perceived effort, efficacy, and their interaction, which could influence the well-being of autistic people in both positive and negative ways. To assess these factors, we modified the Compensation Checklist [15], which was developed through qualitative accounts of autistic people on the use of social strategies, focusing on concrete behaviors [16]. This addresses a limitation of the widely used CAT-Q, which includes items that assess both the use of social strategies and the psychological pressures they impose, potentially confounding the strategies themselves with the psychological toll they take and biasing results toward negative outcomes. In the Compensation Checklist, participants are asked if they use any of the 31 strategies by responding with a yes/no answer, thus capturing a wide range of social strategies used by autistic people. These strategies are conceptually divided into four types: masking (regulating pre-existing social behaviors: e.g., suppressing behaviors such as hand flapping or fidgeting); shallow compensation (displaying neurotypical behaviors without addressing cognitive differences: e.g., looking at the bridge of the nose of the other person with whom they are interacting instead of into their eyes); deep compensation (resolving cognitive differences through alternative means: e.g., learning nonverbal cues to infer others’ thoughts/feelings); and accommodation (using others or the environment to better accommodate oneself without altering oneself: e.g., disclosing a difficulty or diagnosis to receive better accommodation). These four types of strategies are predicted to have different effects on well-being [17]. For instance, masking has been reported to lead to burnout, a loss of sense of true self [18], and emotional and physical distress [19]. Deep compensation is considered more flexible and less taxing than shallow compensation and thus less associated with poor mental well-being [5,17]. Accommodation is predicted to facilitate understanding of others and thus improve well-being [5,17]. However, no study has empirically examined the impact of all four strategies identified by the Compensation Checklist on well-being.

In the current study, we aim to address a gap in knowledge that has not been examined in previous studies: how self-perceived efficacy and the balance between effort and efficacy in the use of social strategies by autistic people, in addition to self-perceived effort, influence their well-being. To do so, we modified the Compensation Checklist by adding questions that explicitly assess both self-perceived effort and efficacy for each strategy, an approach not previously explored, to create the Modified Compensation Checklist (MCC). We propose three hypotheses: (1) High self-perceived effort, which reflects the cognitive burden and psychological stress associated with these strategies, has a negative impact on autistic people’s well-being. (2) High self-perceived efficacy, which reflects the practical benefits of social strategies, has a positive impact on autistic people’s well-being. (3) A balance between self-perceived effort and efficacy affects autistic people’s well-being. In other words, we hypothesize that the interaction between self-perceived effort and efficacy (i.e., “cost-effectiveness” or high efficacy achieved with relatively low effort) is positively associated with autistic people’s well-being. In addition to testing these three hypotheses, we explore whether the different types of social strategies described in the Compensation Checklist affect autistic people’s well-being. To test the hypotheses, we conducted an online survey using the MCC. Self-declared autistic participants took part in this online survey. The effects of self-perceived effort, efficacy, and their interaction effect on self-reported well-being were analyzed using multiple regression.

## 2. Materials and Methods

### 2.1. Participants

Participants were autistic people between the ages of 18 and 65 who lived in Japan and self-reported a diagnosis of autism. Between January and July 2023, a total of 90,000 individuals were initially contacted through an online survey company (iBRIDGE, Inc., Osaka, Japan). After screening for self-reported autism diagnoses and excluding inattentive respondents, 104 participants were included in the final analysis (female: *n* = 48; male: *n* = 56; age: M = 36.1 years, SD = 10.8), exceeding the target sample size of 84. The sample size calculation was based on the previous study, which examined the relationship between autistic people’s social strategies and their well-being [20] (see Methods S1 for details). Detailed demographic information is provided in Table 1.

### 2.2. Screening Procedure

A two-step survey protocol was implemented to screen for participants with a self-reported autism diagnosis and to exclude those who engaged in satisficing (i.e., not paying sufficient attention to the survey), a common problem in online surveys [21] (see Methods S2 and Figure S1 for details). The overall structure of the survey is shown in Figure S2.

The first step was a pre-screening survey to identify participants who self-reported an autism diagnosis. The second step was the main survey, in which participants completed the questionnaires examined in this study. The survey included items designed to detect satisficing, such as the Instructional Manipulation Check (IMC) [22] (see Methods S3 for items used in this study) and the Directed Questions Scale (DQS) [23] (see Methods S4 for items used in this study).

### 2.3. Ethical Approval and Participant Consent

Ethical approval for this study was obtained from the Ethics Committee of Hamamatsu University School of Medicine (#22-171), and the study was conducted following the Declaration of Helsinki [24]. Before answering the questionnaire, participants were presented with an information sheet and the option to complete a consent form. Only those who completed the consent form had access to the survey.

### 2.4. Measures

#### 2.4.1. Modified Compensation Checklist (MCC)

The Compensation Checklist [16], comprising items that describe social strategies used by autistic people, has four subscales: masking, shallow compensation, deep compensation, and accommodation. To evaluate the self-perceived effort and efficacy of autistic people’s social strategies, the items on the Compensation Checklist were modified using the following three steps. The full text of MCC and the changes made by the authors, along with detailed explanations of the changes and their rationale, can be found in Methods S5.

First, the items on the Compensation Checklist were translated from English to Japanese and then back-translated by an academic translation company (Crimson Interaction Pvt. Ltd., Mumbai, India). Second, after translation, the authors modified some of the items to make them easier for participants to understand by shortening them and/or providing examples (9 items; the number of edited items may overlap between different reasons for editing), focusing on concrete behaviors and reducing ambiguity to better align with the study’s objectives (3 items), and adapting them to fit Japanese cultural contexts (7 items; e.g., “rolling one’s eyes” as a sign of boredom has been changed to “yawning during conversation”, as rolling one’s eyes is not a common non-verbal expression in Japanese social interactions). Third, and most critically, questions that asked about self-perceived effort and efficacy of each strategy were added to the original questions about the use of each strategy.

For each item of the MCC, participants were presented with yes/no questions about whether they used any of the included strategies, which were the questions originally included in the Compensation Checklist (“Do you usually use the following strategies in social situations? Please answer yes or no”). Only if the response was “yes” to using each strategy, were participants then asked about their self-perceived effort (“Has it been tiring/difficult for you to use the following strategies in social situations? Please answer the choice that best applies to you”) and self-perceived efficacy (“Has using the following strategies in social situations made it easier for you to live or get along in society? Please answer the choice that best applies to you”) were presented. These newly added questions were to be answered on a scale ranging from 1 (“strongly disagree”) to 7 (“strongly agree”) for the strategies they reported using, with higher scores indicating greater effort and efficacy.

#### 2.4.2. The Japanese Version of the Warwick-Edinburgh Mental Wellbeing Scale (WEMWBS)

The WEMWBS [25] is a 14-item self-report questionnaire that measures general well-being over the previous two weeks of the survey (e.g., I’ve been feeling relaxed). The WEMWBS has been translated into Japanese [26] and has been widely used in autism research, including many studies examining social strategies (e.g., [7,27]). Each response ranges from 1 (none of the time) to 5 (all of the time), with higher scores indicating greater overall well-being.

#### 2.4.3. The Japanese Version of the UCLA Loneliness Scale Version 3 (UCLA-LS)

The UCLA-LS [28] is a 20-item self-report questionnaire that measures loneliness with responses ranging from 1 (never) to 4 (always), with higher scores indicating greater perceived loneliness. Because the WEMWBS measures general well-being and contains few items directly relating to social interactions, the UCLA-LS was used as a secondary measure to assess social well-being. The UCLA-LS contains items enquiring about social well-being in everyday life (e.g., how often do you feel part of a group or friends), as opposed to most scales that measure social well-being, where “social” is used in the sense of political or societal (see [29] for a review). The UCLA-LS is considered to capture the loneliness of autistic people accurately [30], and it has been translated and validated in Japanese [31].

#### 2.4.4. The Japanese Version of the Autism Spectrum Quotient (AQ)

The AQ [32] is a 50-item self-report questionnaire widely used to assess autistic traits (e.g., I prefer to do things the same way over and over again), and it has been translated into Japanese (AQ) [33]. Responses are scored binarily (0: “Definitely Disagree”, “Slightly Disagree”; and 1: “Slightly Agree”, “Definitely Agree”), with higher scores indicating higher levels of autistic traits. The AQ was used to ensure the validity of the autistic traits of participants gathered online during the pre-screening.

### 2.5. Analyses

Linear regression models were used to test the hypotheses that autistic people’s self-perceived effort and efficacy in using social strategies and their interaction effect are associated with their well-being. In these models, the total scores of WEMWBS and UCLA-LS were included as dependent variables, and self-perceived effort and efficacy of the strategies used and the interaction between effort and efficacy, which was obtained from the MCC, were included as independent variables. In addition, age, sex, and number of strategies used were included as controlling variables. Each participant differed in the number of strategies used; hence, effort and efficacy scores were averaged within individuals in these models. Furthermore, to gain a better understanding of the observed results of the interaction effect, self-perceived efficacy was plotted against self-perceived effort. A linear regression with quadratic terms was then performed to obtain a better characterization of the relationship between self-perceived effort and efficacy.

In addition to the analysis to test our initial hypothesis, we conducted the following two exploratory analyses. First, we examined whether the main effect of self-perceived effort and efficacy of social strategy used, as well as their interaction effect, was associated with well-being when examining individual MCC subscales (masking, shallow compensation, deep compensation, assimilation). The linear regression models similar to those in the primary analysis were examined, but by each subscale. Participants who reported using none of the strategies in any of the individual subscales were excluded from the analysis. For the model in which there was a significant interaction effect, a simple slope analysis was performed to determine the direction of the effect.

Second, to examine the association between well-being and the number of strategies autistic people chose to use in each MCC subscale (as opposed to the averaged self-perceived effort and efficacy of used strategies), linear regression was conducted with WEMWBS and UCLA-LS scores as dependent variables and binary strategy use (yes/no) by subscale, in addition to age and sex as independent variables.

All analyses were performed using R (version 4.1.1) [34] with the following additional packages: “tidyverse” [35] for overall data processing and visualization, “jtools” for visualizing the coefficients of linear regressions [36], and “interactions” for performing and visualizing simple slope analysis for a significant variable found in linear regression [37].

## 3. Results

### 3.1. Linear Regression of Association between WEMWBS/UCLA-LS and Self-Perceived Effort and Efficacy of Used Social Strategies

**Figure 1:**
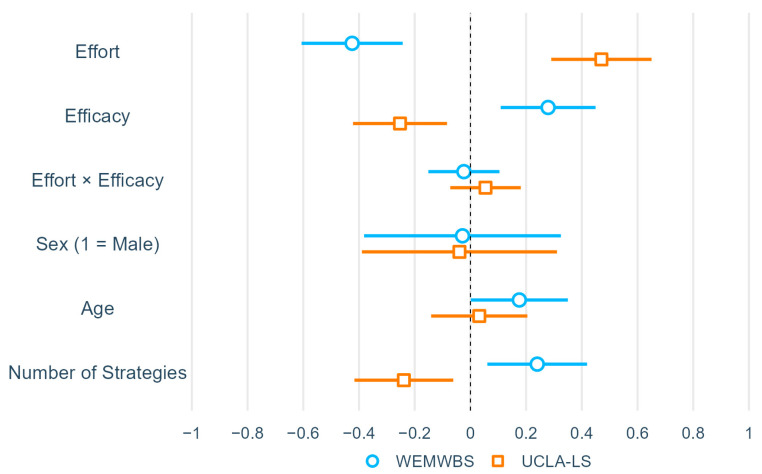
Visualization of regression of association between WEMWBS/UCLA-LS and self-perceived effort and efficacy of social strategies used. *Note:* N = 104. Bars represent 95% confidence intervals.

Two linear regression models were used to examine the main effects of and interactions between self-perceived effort and efficacy of social strategies on WEMWBS and UCLA-LS (Table 2; for visualization of coefficients, see Figure 1). Both models were significant and demonstrated large effect sizes (WEMWBS: *R^2^* = 0.281, *p* < 0.001; UCLA-LS: *R^2^* = 0.294, *p* < 0.001; the practical significance of effect sizes is interpreted based on conventions of behavioral science [38]). The main effects of self-perceived effort (WEMWBS: *β* = −0.42, *p* < 0.001; UCLA-LS: *β* = 0.47, *p* < 0.001) and efficacy (WEMWBS: *β* = 0.28, *p* = 0.002; UCLA-LS: *β* = −0.25, *p* = 0.004) were significant. The interaction effect between effort and efficacy was, by contrast, not significant in either model (WEMWBS: *β* = −0.02, *p* = 0.717; UCLA: *β* = 0.05, *p* = 0.397). A weak quadratic trend was observed in the relationship between self-perceived effort and efficacy (*R^2^* = 0.07, *p* = 0.010; Figure S3).

In addition, the effect of the number of strategies was significant in both models (WEMWBS: *β* = 0.24, *p* = 0.009; UCLA-LS: *β* = −0.24, *p* = 0.009). The effect of age was significant only in the WEMWBS (*β* = 0.18, *p* = 0.048). The effect of sex was not significant in either model.

To further validate these findings, we conducted the same analyses, this time excluding participants who self-reported a clinical diagnosis of other neurodevelopmental disorders, for both the WEMWBS (Table S1) and the UCLA-LS (Table S2); thus excluding (1) participants with Intellectual Developmental Disorder (IDD; *n* = 6), (2) participants with Specific Learning Disorder (SLD; *n* = 5), and (3) participants with IDD and/or SLD (*n* = 8). All models showed large effect sizes. For the WEMWBS, the results were largely consistent with the primary analysis, indicating significant main effects for effort and efficacy, except for (a) the effect of the number of strategies, which was not significant when excluding participants with IDD (*β* = 0.18, *p* = 0.056) and participants with IDD and/or SLD (*β* = 0.16, *p* = 0.095), and (b) the effect of age, which was not significant in any model that excluded participants with comorbidities (IDD: *β* = 0.18, *p* = 0.051; SLD: *β* = 0.14, *p* = 0.119; IDD and/or SLD: *β* = 0.17, *p* = 0.055). For the UCLA-LS, the results were mostly consistent with the primary analysis, including the main effects of effort, efficacy, and the number of strategies, except for the effect of age, which was not significant for any model that excluded participants with other neurodevelopmental comorbidities (IDD: *β* = 0.04, *p* = 0.689; SLD: *β* = 0.06, *p* = 0.463; *β* = 0.05, *p* = 0.565).

### 3.2. Linear Regression of Association between WEMWBS/UCLA-LS and Self-Perceived Effort and Efficacy of Social Strategies Used in Each MCC Subscale

Eight linear regression models were used to examine the main and interaction effects of self-perceived effort and efficacy of social strategies on WEMWBS and UCLA-LS in each MCC subscale: masking, shallow compensation, deep compensation, and accommodation (Tables S3 and S4; for visualization of coefficients, see Figures S4 and S5). All models were significant and presented medium effect sizes, and for both WEMWBS and UCLA-LS, the main effect of effort was significant in most models, including masking, shallow compensation, and deep compensation, except for accommodation in WEMWBS (*β* = −0.18, *p* = 0.073). Similarly, the main effect of efficacy was significant in most models, including masking, deep compensation, and accommodation, except for shallow compensation in UCLA-LS (*β* = −0.14, *p* = 0.154).

A significant interaction effect was observed only in the association between deep compensation and WEMWBS (*β* = −0.17, *p* = 0.044). To further interpret this interaction, simple slope analyses were conducted to examine the effect of efficacy at three different levels of effort: the mean, one standard deviation above the mean, and one standard deviation below the mean (Figure S6). The slope of efficacy was significantly positive at one standard deviation below the mean (*β* = 0.52, *p* = 0.002) and at the mean (*β* = 0.35, *p* = 0.002), whereas it was not significant at one standard deviation above the mean (*β* = 0.188, *p* = 0.068).

In addition, the effect of the number of strategies was significant in deep compensation for WEMWBS (*β* = 0.30, *p* = 0.003), in shallow compensation (*β* = −0.22, *p* = 0.026), and in deep compensation for UCLA-LS (*β* = −0.22, *p* = 0.030). The effect of age was significant in shallow compensation (*β* = 0.22, *p* = 0.030), deep compensation (*β* = 0.28, *p* = 0.005), and accommodation (*β* = 0.20, *p* = 0.049) for the WEMWBS but not for the UCLA-LS. The effect of sex was not significant in any of the models.

### 3.3. Linear Regression of Association between WEMWBS/UCLA-LS and the Use of Social Strategies in Each MCC Subscale

**Figure 2:**
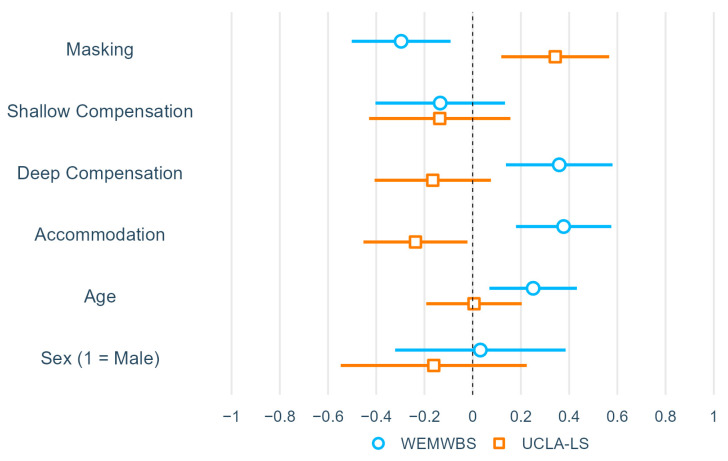
Visualization of regression of association between WEMWBS/UCLA-LS and the use of MCC subscale strategies. *Note:* N = 104. Bars represent 95% confidence intervals.

Two linear regression models were used to examine the relationship between the number of social strategies within each MCC subscale that each participant used and the WEMWBS and UCLA-LS (Table 3; for visualization of coefficients, see Figure 2). Both models were significant, showing large and medium effect sizes (WEMWBS: *R*^2^ = 0.273, *p* < 0.001; UCLA-LS: *R*^2^ = 0.134, *p* = 0.003). The number of masking strategies used was significant and negatively associated with the WEMWBS (*β* = −0.30, *p* = 0.005) and positively associated with the UCLA-LS (*β* = 0.34, *p* = 0.003). The number of shallow compensation strategies used was not significantly associated with WEMWBS (*β* = −0.13, *p* = 0.322) or UCLA-LS (*β* = −0.14, *p* = 0.357). The number of deep compensation strategies used was significantly associated with the WEMWBS (*β* = 0.36, *p* = 0.002) but not with the UCLA-LS (*β* = −0.17, *p* = 0.176). The number of accommodation strategies used was significantly associated with the WEMWBS and UCLA-LS, but the direction of the association was opposite to that of the other subtypes. It was positively associated with WEMWBS (*β* = 0.38, *p* < 0.001) and negatively associated with UCLA-LS (*β* = −0.24, *p* = 0.031). In addition, older age was significantly associated with higher WEMWBS scores (*β* = 0.25, *p* = 0.007). The effect of sex was not significant in either model.

## 4. Discussion

This is the first study to examine how autistic people’s use of social strategies affects their well-being, by focusing on both self-perceived effort and efficacy in using social strategies, as well as the interaction effect between them. By modifying the Compensation Checklist, this study demonstrated that self-perceived effort in using social strategies was negatively associated with autistic people’s well-being, whereas self-perceived efficacy was positively associated with their well-being. Importantly, all of the models analyzed had medium to large effect sizes; in particular, the models in the main analysis had large effect sizes. This suggests that the associations are not only statistically significant but also practically meaningful, highlighting the substantial impact of the use of social strategies on the well-being of autistic people. Furthermore, these results were corroborated by a measure of loneliness, which is considered to reflect the social aspect of well-being, showing an inverse relationship to well-being. In addition, sensitivity analyses excluding participants with IDD and/or SLD showed that the effects of both self-perceived effort and efficacy closely mirrored those of the main analyses, indicating the robustness of these findings.

A high self-perceived effort in the use of social strategies negatively affected the well-being of autistic people. This finding is consistent with previous qualitative studies reporting that use of social strategies contributes to burnout, low self-esteem, exhaustion [4], and loneliness [6]. Conversely, high self-perceived efficacy in using social strategies was positively associated with the well-being of autistic people. This finding is in line with qualitative accounts from autistic people who report that using social strategies can help them navigate social interaction, manage impressions of others, and act as a defense mechanism to avoid stressful or uncomfortable situations [3,4,10]. By introducing the concepts of self-perceived effort and efficacy of social strategies, we qualitatively demonstrated the double-edged nature of social strategies on autistic people’s well-being for the first time, which was highlighted by previous qualitative studies [3,4,10]. Therefore, instead of focusing on the use or non-use of social strategies, we suggest that it is imperative to consider both the potential practical benefits and the psychological burdens of social strategies to improve the well-being of autistic people. This implication echoes the call for clinicians to adopt a more nuanced approach that considers both the costs and benefits of using social strategies to improve well-being [3]. Interventions that adopt this nuanced approach, incorporating the self-perceived effort and efficacy of social strategies, can help autistic people become more mindful of the strategies they use, potentially reducing harm and enhancing the benefits, thereby improving their overall well-being. And more practically, the study highlights the need for a more inclusive society, where autistic people can more easily opt out of effortful social strategies and focus on more efficient ones. This is because, under the current societal context, autistic people often feel compelled to use certain social strategies to ensure their social safety [3], even though it might involve greater self-perceived effort and yield less self-perceived efficacy. Moreover, the negative association between self-perceived effort and the well-being of autistic people was also consistent with findings from previous studies using the CAT-Q, which found similar associations between self-perceived efforts and lower well-being manifesting as depression, anxiety, and stress [8,9]. Therefore, it can be inferred that these previous studies using CAT-Q primarily reflected a similar aspect of the self-perceived effort of using social strategies.

In addition to examining the main effects of self-perceived effort and efficacy of using social strategies, we investigated their interaction effect on autistic people’s well-being. We hypothesized that the interaction effect between self-perceived effort and efficacy, i.e., low self-perceived effort and high self-perceived efficacy when using social strategies, would be positively associated with autistic people’s well-being, but the interaction effect was not significant. Subsequent exploratory analyses that examined the relationship between well-being and the interaction effects on each strategy type also confirmed the overall pattern of these results. These findings suggest that the “cost effectiveness” of self-perceived efficacy relative to self-perceived effort is not uniquely associated with autistic people’s well-being, and thus focusing on this “cost effectiveness” alone may not benefit their well-being. One possible explanation for this is that the variance of the interaction term was small due to the quadratic distribution between self-perceived effort and efficacy (Figure S3). This distribution implies that self-perceived effort and efficacy are not always in “balance”, and some feel more efficacy while perceiving less effort, and vice versa. It is possible that those who invest in social strategies either selectively and with minimal effort, or a lot of effort, may experience large benefits, while those who invest in social strategies moderately may not benefit much. Another possible reason why the interaction effect was not significant could be that it was diminished when all types of strategies were analyzed together. When only deep compensation was examined, a significant interaction effect between effort and efficacy emerged, suggesting an effect of “cost effectiveness.” Moreover, the observed trend in the coefficients of each type of strategy was consistent for both well-being and loneliness, with deep compensation showing the largest effect size, followed by shallow compensation, accommodation, and finally masking. Although accommodation differs from the other strategies in that it involves external factors such as other people and the environment, when comparing the other strategies, one could argue that deep compensation is a more complex and versatile strategy and is more adaptable to different social contexts. In contrast, shallow compensation can be effective but less sophisticated, and masking is the most basic and least flexible. This difference may explain why the more sophisticated strategy, such as deep compensation, is influenced by cost-effectiveness, while simpler strategies show reduced effect sizes in the interaction effect. This finding also draws an interesting parallel with reports from autistic people describing changes in social strategies over time; some autistic people became more selective in their use of strategies depending on social contexts [5], which may suggest that some autistic people may acquire optimal ways of using social strategies that minimize effort and maximize efficacy over time.

In addition to self-perceived effort and efficacy, the number of strategies that each autistic person uses in their daily life is another important factor for their well-being, an aspect that was not examined previously. Interestingly, the number of strategies used was positively associated with well-being and negatively associated with loneliness. The ability to use a wider range of social strategies may provide the ability to adapt to different social situations flexibly, helping autistic people with social interactions, and may improve their well-being. This implication is also suggested by autistic people describing the course of development of their use of social strategies, the different social strategies they learned over time, the decision to use particular strategies, and the choice of strategy they made in particular social situations [5]. However, this effect was not significant in the sensitivity analyses that excluded participants with IDD and/or SLD, and, therefore, this finding should be interpreted with caution.

Exploratory analyses revealed that each of the four types of social strategies identified in the Compensation Checklist [15] was differently associated with well-being. First, masking was associated with decreased well-being and increased loneliness, consistent with previous theoretical and qualitative research suggesting that conscious suppression of innate autistic behaviors leads to burnout and a loss of sense of true self [18] and an emotional and physical toll [19]. Second and third, shallow compensation was not significantly associated with either well-being or loneliness, but deep compensation was positively associated with well-being but not with loneliness. This suggests that actively engaging in superficial behaviors to compensate for social difficulties is neither beneficial nor detrimental to well-being, but understanding and acquiring the cognitive skills involved can improve well-being. This finding supports the assumption that deep compensation is more automatic, flexible, sophisticated, and less cognitively taxing than shallow compensation [5,17]. Fourth, accommodation was associated with increased well-being and decreased loneliness, thus suggesting that strategies involving others and the environment can improve autistic people’s well-being. This is consistent with previous studies that have theorized the positive effects of accommodation [5,17]. Taken together, approaches that encourage the use of, or the shift toward, deep compensation and accommodation strategies from masking strategies may improve the well-being of autistic people. Concurrently, societal interventions are crucial to reduce stigma and pressure on autistic people, facilitating this transition.

Although these results are compelling, four limitations need to be acknowledged. First, this study employed a novel recruitment approach by sourcing autistic participants from an online survey service pool, which included individuals irrespective of their diagnostic status (i.e., including non-autistic people), who were later screened based on self-reported autism diagnoses. This method differs from the majority of prior studies that typically recruit participants from self-advocacy groups, autism-related organizations, universities, hospitals, or through advertisements specifically targeting autistic people. While the current approach has the advantage of reaching autistic people unaffiliated with specific organizations—potentially offering more representative participants of the broader autistic population—it is recommended that future studies replicate these findings with participants recruited through more traditional methods to confirm the generalizability. Second, although we believe the modifications made to adapt the original Compensation Checklist to the Japanese context were minor and did not alter the core elements of the measure, the generalizability of these findings to other cultural contexts remains an open question. Therefore, future studies should aim to replicate these findings in different cultural settings. Third, this study did not fully account for all the possible confounders of the relationship between social strategies and well-being in autistic people, due to practical limitations. Particularly, addressing demographic factors such as socioeconomic status [39] and social support [40], which are known to influence well-being—higher socioeconomic status and stronger social support are associated with greater well-being and vice versa—would benefit future studies to provide a more comprehensive understanding. Fourth, this study relied exclusively on self-report measures to assess the self-perceived effort and efficacy of social strategies and the well-being of autistic people. While self-report questionnaires are valuable for capturing personal experiences and subjective perceptions, they are also subject to biases. For example, negative experiences are generally more salient and easier to remember than positive ones [41], which may lead to an inflation of reported self-perceived effort. In addition, self-report measures can be influenced by other factors such as social desirability bias and recall inaccuracies. Future research should consider incorporating complementary objective measures, such as third-party reports or behavioral observations, to provide a more comprehensive understanding of social strategy use and its impact on well-being.

In addition to addressing these limitations, future research can further explore how different social contexts influence the self-perceived effort and efficacy of social strategies. For example, shallow or deep compensation may provide satisfactory levels of efficacy in brief encounters while minimizing effort, whereas accommodation strategies may provide greater efficacy in long-term settings, such as the workplace, despite requiring more initial effort. In addition, social environments vary in their level of knowledge about autism, attitudes toward neurodiversity, and acceptance toward non-neurotypical behavior—all of which may influence how autistic people use social strategies. Incorporating experience sampling methods to assess effort and efficacy in a variety of real-life social situations may provide a more comprehensive understanding of the relationship between social strategies and the well-being of autistic people.

## 5. Conclusions

The current study demonstrated the double-edged nature of autistic people’s use of social strategies in everyday life, in that it has both positive and negative impacts on their well-being. These findings suggest the importance of considering both the potential practical benefits of using social strategies as well as their psychological burden on autistic people to improve their well-being. Additionally, the results suggest that having a wider repertoire of social strategies may enhance the well-being of autistic people. Moreover, this study found that different types of social strategies, such as masking, shallow compensation, deep compensation, and accommodation, have varied effects on autistic people’s well-being. The findings of this study call for a more nuanced discussion among researchers and clinicians regarding the social strategies used by autistic people, rather than simply encouraging or discouraging their use entirely.

## Figures and Tables

**Table 1:** Participants’ characteristics.

Characteristic	Overall (*n* = 104)	Female (*n* = 48)	Male (*n* = 56)	*p*
Age (SD)	36.1 (10.8)	33.5 (9.8)	38.4 (11.1)	0.021 ^a^
AQ (SD)	33.6 (7.0)	34.4 (6.8)	32.9 (7.1)	0.281 ^a^
Comorbidities (%)	26 (25%)	14 (29%)	12 (21%)	0.364 ^b^
ADHD	24 (23%)	12 (25%)	12 (21%)	0.667 ^c^
SLD	5 (4.8%)	2 (4.2%)	3 (5.4%)	1.000 ^c^
IDD	6 (5.8%)	3 (6.3%)	3 (5.4%)	1.000 ^c^

*Note:* ADHD = Attention-Deficit/Hyperactivity Disorder. SLD = Specific Learning Disorder. IDD = Intellectual or Developmental Disorder. ^a^ Wilcoxon rank sum test; ^b^ Pearson’s chi-squared test; ^c^ Fisher’s exact test.

**Table 2:** Regression of association between WEMWBS/UCLA-LS and self-perceived effort and efficacy of social strategies used.

Variables	WEMWBS				UCLA-LS			
	*β*	95% CI		*p*	*β*	95% CI		*p*
		*LL*	*UL*			*LL*	*UL*	
Effort	−0.42	−0.61	−0.24	<0.001	0.47	0.29	0.65	<0.001
Efficacy	0.28	0.11	0.45	0.002	−0.25	−0.42	−0.08	0.004
Effort × Efficacy	−0.02	−0.15	0.10	0.717	0.05	−0.07	0.18	0.397
Sex ^a^	−0.03	−0.38	0.32	0.874	−0.04	−0.39	0.31	0.824
Age	0.18	0.00	0.35	0.048	0.03	−0.14	0.20	0.715
Number of Strategies	0.24	0.06	0.42	0.009	−0.24	−0.42	−0.06	0.009
Adjusted *R*^2^	0.281				0.294			
*p*	<0.001				<0.001			

*Note:* N = 104. CI = confidence interval; *LL* = lower limit; *UL* = upper limit. ^a^ 0 = female, 1 = male.

**Table 3:** Regression of association between WEMWBS/UCLA-LS and the use of MCC subscale strategies.

Variables	WEMWBS				UCLA-LS			
	*β*	95% CI		*p*	*β*	95% CI		*p*
		*LL*	*UL*			*LL*	*UL*	
Masking	−0.30	−0.50	−0.09	0.005	0.34	0.12	0.57	0.003
Shallow Compensation	−0.13	−0.40	0.13	0.322	−0.14	−0.43	0.16	0.357
Deep Compensation	0.36	0.14	0.58	0.002	−0.17	−0.41	0.08	0.176
Accommodation	0.38	0.18	0.58	<0.001	−0.24	−0.45	−0.02	0.031
Age	0.25	0.07	0.43	0.007	0.01	−0.19	0.20	0.956
Sex ^a^	0.03	−0.32	0.39	0.859	−0.16	−0.55	0.22	0.408
Adjusted *R*^2^	0.273				0.134			
*p*	<0.001				0.003			

*Note:* N = 104. CI = confidence interval; *LL* = lower limit; *UL* = upper limit. ^a^ 0 = female, 1 = male.

## Data Availability

The raw data supporting the conclusions of this article will be made available by the authors on request due to privacy.

## Supplementary Materials

The following supporting information can be downloaded at: https://www.mdpi.com/article/10.3390/brainsci14100962/s1, Methods S1: Estimation of target participant size; Methods S2: Participant recruitment and screening procedure; Methods S3: Instructional Manipulation Check; Methods S4: Directed Questions Scale; Methods S5: Modified Compensation Checklist; Figure S1: Flowchart of participant recruitment and screening procedure; Figure S2: Survey structure; Figure S3: Distribution of self-perceived effort and efficacy of using social strategies; Figure S4: Visualization of linear regression of association between WEMWBS and self-perceived effort and efficacy of social strategies used, in each MCC subscale; Figure S5: Visualization of linear regression of association between UCLA and self-perceived effort and efficacy of social strategies used, in each MCC subscale; Figure S6: Interaction effect of self-perceived and efficacy using deep compensation strategies in WEMWBS; Table S1: Regression of association between WEMWBS and self-perceived effort and efficacy, excluding participants with IDD and/or SLD; Table S2: Regression of association between UCLA-LS and self-perceived effort and efficacy, excluding participants with IDD and/or SLD; Table S3: Regression of association between WEMWBS and self-perceived effort and efficacy, excluding participants with IDD and/or SLD; Table S4: Regression of association between UCLA-LS and self-perceived effort and efficacy, excluding participants with IDD and/or SLD. References [42,43,44,45,46,47,48] are cited in the Supplementary Materials.
