# Supplementary material for: Double-Edged Effects of Social Strategies on the Well-Being of Autistic People: Impact of Self-Perceived Effort and Efficacy
Source: Brain Sci. 2024 Sep 25;14(10):962. doi: 10.3390/brainsci14100962 (PMC11506214; doi:10.3390/brainsci14100962)
Supplement: Supplementary file 1 [file brainsci-14-00962-s001.zip › brainsci-3195316-supplementary.pdf]

Methods S1

Estimation of Target Participant Size

The target participant size was estimated based on previous research and the proposed model for the hypothesis. Given the absence of prior studies measuring both the self-perceived effort and efficacy of autistic people's social strategies, approximate effect sizes were borrowed from a study on the similar study examining the association between multiple mental health outcomes and social strategies of autistic people [20], adopting the most conservative effect size observed in that study (R^2 = 0.15). In planning a linear regression with six dependent variables and following the standard convention of behavioral science for alpha and power ($\alpha = 0.05$, $1 - \beta = 0.80$; [42]), the target sample size was estimated to be 84 using G*Power [43, 44]. A total of 104 participants were included in the analysis, surpassing the estimated target participant size.

Methods S2

Participant Recruitment and Screening Procedure

The participant recruitment was conducted in three phases to achieve the target sample size who meet the inclusion criteria (see Figure S1). In the first phase, 10,000 participants were recruited for pre-screening. Participants completed the survey from any location and device and were compensated based on the rate provided by the survey company. Participants were asked to self-report if they had received a clinical diagnosis of autism, and only those who self-reported to have received a clinical diagnosis of autism were administered the main survey. In addition to the pre-screening, the main survey also had screening questions consisting of two parts; (1) re-screening for clinical autism diagnosis, in which participants re-reported their clinical diagnosis of autism to confirm consistency with their initial report, and (2) detection for satisficing.

Satisficing is a behavior in which participants do not pay enough attention to the survey items to conserve their attentional resources [45]. It is known to be particularly prevalent in online questionnaire-based research [21] and is known to undermine the validity of research. To detect participants who engage in satisficing, one Instructional Manipulation Check (IMC) [22] and two Directed Questions Scales (DQS) [23] were incorporated into the survey. The IMC is a question designed to detect respondents who do not read the directions in the questionnaire carefully. In the IMC, a dummy direction is presented along with the corresponding items, but in the later part of the direction, respondents are asked to perform a specific action instead of answering questions normally (e.g., “If you have read these instructions, please do not answer any of the following questions [i.e., do not select any of the options] and proceed to the next page”). DQS is a question designed to detect respondents who do not properly read items. DQS directly asks participants to perform a specific action for a given item (e.g., “For this question, answer ‘Never’”). All IMC and DQS items used in this study are available in Methods S3 and S4, respectively.

Participants who were inconsistent with their self-reported autism diagnosis on the pre-screening and main survey questions (e.g., those who reported having an autism diagnosis in the pre-screening but did not do so in the main survey) or who exhibited satisficing behaviors identified by the IMC and DQS were excluded from the analysis. Of the 334 participants who passed the pre-screening, only 25 met all the criteria required for inclusion. The extremely high exclusion rate was attributed to inconsistencies in reported autism diagnoses and detection of satisficing, both of which most likely indicate satisficing.

In the second phase, the pre-screening process was tightened by adding measures to detect satisficing to address the extremely high exclusion rate. These additional measures included 1 IMC and 2 DQS items, the same number of items incorporated in the main survey, as well as dummy questions from other existing questionnaires, such as the Japanese version of the Bermond-Vorst Alexithymia Questionnaire [46], the Integrating Conflict Resolution Skills Scale [47], and the

Japanese version of the World Health Organization Quality of Life [48]. Nine items were extracted from each of these questionnaires and used as dummy questions. In this phase, only those participants who self-reported to have an autism diagnosis and passed all the IMC and DQS were invited for the main survey. Participants who completed the main survey were then excluded from the analysis based on the same criteria as before; those who reported inconsistent self-report of clinical diagnosis of autism on the pre-screening and main survey, and those who exhibited satisficing as determined by the IMC and DQS. A total of 30,000 participants were recruited for pre-screening, of whom 22 were included in the final analysis.

Due to the very high exclusion rate still observed in the second phase, an additional 50,000 participants were recruited for pre-screening in the third phase, which used the same protocol as the second phase. Out of those participants, 57 met all the inclusion criteria. In total, 90,000 Japanese participants were recruited for pre-screening, of whom 104 autistic participants met all the inclusion criteria and were included in the analysis.

Methods S3

Instructional Manipulation Check (IMC; based on [22])

Both of the IMC questions were created originally. Instructions to detect satisficing are bolded and underlined for the ease of readers. IMC 1 and 2 were included in the pre-screening, along with dummy questions. IMC 3 was included in the main survey. For details regarding the presentation and placements of IMC, refer to Figure S2. The original (Japanese) text is appended below the translated text.

IMC 1

In recent years, the need to learn foreign languages has increased due to the rapid progress of globalization. On the other hand, with the development of AI, the accuracy of machine translation is increasing, making it easier for people who were previously unfamiliar with foreign languages to interact with people from other countries and use foreign services. In other words, the importance of language itself is being recognized as more attention is paid to foreign languages. In our research, we also try to test new hypotheses based on the responses we get from you when you read the written language. Therefore, we are interested in the extent to which you have read the text written here. **If you have read this paragraph, please answer "disagree" to all of the following questions.** Thank you for your cooperation.

Options:

1. Strongly disagree
2. Disagree
3. Somewhat disagree
4. Neither agree nor disagree
5. Somewhat agree
6. Agree
7. Strongly agree

Questions (dummy):

1. It is beneficial for me to learn a foreign language.
2. Learning a foreign language broadens my horizons.
3. Learning a foreign language reduces discrimination and prejudice
4. Learning a foreign language is difficult for me.
5. To learn a foreign language, you need to study where the language is spoken.
6. To learn a foreign language, it is important to speak with native speakers of the language.

7. To learn a foreign language, it is necessary to have explanations in Japanese (or other native languages).
8. Learning a foreign language is fun.
9. Being able to speak a foreign language is more important than being able to write a foreign language.
10. I am embarrassed to practice speaking a foreign language out loud.

IMC 2

We would like to ask you about you and the Internet. Web-based surveys are increasingly being used in research on the social behavior of autistic people. In recent years, the Internet has become an essential part of autistic people's lives. Many autistic people connect with other autistic people through the Internet, and their way of life has changed dramatically with the promotion of working from home or online courses due to the coronavirus. We are also conducting a web-based survey of autistic people's lives, but some problems have been pointed out with this method as well. That is, we have no way of knowing how people are responding to the survey, and we do not know if they are reading the explanations and choices properly, without skipping over them. Therefore, we are also interested in whether you read this text. **If you have read these instructions, please do not answer any of the following questions (i.e., do not select any of the options) and proceed to the next page.** Thank you for your cooperation.

Options:

1. Agree
2. Somewhat agree
3. Neither agree nor disagree
4. Somewhat disagree
5. Disagree

Questions (dummy):

1. I use the Internet regularly.
2. The Internet has improved the quality of life for autistic people.
3. The Internet has made it easier for us to connect with other autistic people.
4. I use the Internet to work from home or take online courses.
5. I use the Internet to gather information about developmental disabilities.
6. I consult professional services (hospital, social services, etc.) using the Internet.
7. My quality of life has improved thanks to the Internet.
8. I encounter problems using the Internet.

9. I can live without the Internet without problems.
10. Social interaction over the Internet feels easier compared to social interaction in person.

IMC 1

近年ではグローバル化が急速に進むことで、外国語を学ぶニーズは高まっていると考えられます。その一方 AI の発展に伴い、機械翻訳の精度が上昇することで、今までは外国語に慣れ親しんでこなかった人でも、外国の人々と関わりを持ったり、外国のサービスを扱ったりしやすくなったりしています。このように、外国語に注目が集まるようになってきたということは、言葉の重要性が強く認識されるようになってきたとも考えられます。私達の研究も、皆様に文章という言葉を読んでいただいて、答えていただいた回答をもとに新しい仮説を検討しようとしています。そこで、皆様がここに書いている文章をどのくらい読んでいらっしゃるのかという点に興味があります。**もしこの文章をお読みになったなら、以下のすべての質問について、「そう思わない」と答えてください。**どうぞよろしくお願いします。

選択肢：
1. まったくそう思わない
2. そう思わない
3. あまりそう思わない
4. どちらでもない
5. ややそう思う
6. そう思う
7. 非常にそう思う

質問（ダミー）：
1. 外国語を学ぶことは、自分にとって有益だと思う
2. 外国語を学ぶことで、視野が広がると思う
3. 外国語を学ぶことで、差別や偏見が減ると思う
4. 外国語を学ぶことは、自分にとって難しい
5. 外国語を学ぶには、その言葉が話されている現地に留学する必要がある
6. 外国語を学ぶ際には、その外国語のネイティブスピーカー（母語話者）と話すことが大切だと思う
7. 外国語を学ぶ際には、日本語（あるいは日本語以外の母語）での解説が必要だと思う
8. 外国語を学ぶのは楽しいことである
9. 外国語を話せるようになることは、書けるようになることよりも重要だと思う
10. 外国語を口に出して練習するのは、恥ずかしいと感じる

IMC 2

あなたのインターネットにかかわる行動についてお尋ねします。ASD 者の社会的行動に関する研究では、インターネットを使った調査が増えてきています。近年ではたくさんの ASD 者がインターネットを通じて他の ASD 当事者とつながったり、コロナウイルス感染症のためにインターネットによる在宅勤務やオンライン授業が普及することで、ASD 者の生活の仕方も大きく変わるなど、ASD 者とインターネットは切っても切れない関係になってきています。私達もインターネットを使って、ASD 者に関する調査を行っていますが、インターネットを使った調査では問題点も指摘されています。それは、回答者の方がどういった状態でご回答をしてくださるのか、説明文や選択肢を読み飛ばさずにきちんと読んで頂いてるのかがわからないということです。そこで私達も、皆様がこの文章を読んでいただいているかについて興味を持っています。**もしこの指示をお読みなったなら、以下の質問には回答せず（つまり、どの選択肢にもチェックを入れずに）次のページに進んでください。**よろしくお願いいたします。

選択肢：

1. あてはまらない
2. あまりあてはまらない
3. どちらともいえない
4. ややあてはまる
5. あてはまる

質問（ダミー）：

1. インターネットをよく使用している
2. インターネットによって、ASD 者の生活は向上したと思う
3. インターネットによって、他の ASD 者と繋がりやすくなったと思う
4. インターネットを使った、在宅勤務やオンライン授業などを活用している
5. インターネットを使って、発達障害に関する情報を収集している
6. インターネットを使って、発達障害に関する相談を専門機関（病院、福祉機関など）にしている
7. インターネットを使うことで、生活の質が向上したと思う
8. インターネットを使うことで、トラブルに遭遇する
9. インターネットがなくても、問題なく生活できると思う
10. インターネットを使った社会的交流は、対面での社会的交流に比べて気楽だと感じる

Methods S4

Directed Questions Scale (DQS; based on [23])

All items were created by the authors. DQS 1 and 2 were included in the pre-screening, and DQS 3 and 4 were included in the main analysis. For details regarding the presentation and placements of DQS, refer to Figure S2. The original (Japanese) text is appended below the translated text.

DQS 1: Please select "Never" for this question.

DQS 2: Please select "Never" for this question.

DQS 3: Please select "6. Agree" for this question.

DQS 4: Please select "2. Slightly agree" for this question.

DQS 1: この質問では、「あてはまらない」と答えてください

DQS 2: この質問には、「まったくない」と答えてください

DQS 3: この質問には、「2. ほとんどない」と答えてください

DQS 4: この質問には、「6. そう思う」と答えてください

Methods S5

Modified Compensation Checklist (MCC; [15])

Participants first answered Question 1 for the list of strategies below and proceeded to Question 2, and then Question 3.

Question 1:

Do you usually use the following strategies in social situations? Please answer Yes or No.

1. Yes
2. No

Question 2 (Displayed only for the items participants answered “Yes” in Question 1):

How tiring or difficult do you find to use the following strategies in social situations? Please answer the choice that best applies to you.

1. Strongly Disagree
2. Disagree
3. Somewhat Disagree
4. Neither Agree nor Disagree
5. Somewhat Agree
6. Agree
7. Strongly Agree

Question 3 (Displayed only for the items participants answered “Yes” in Question 1):

How much do you feel that using the following strategies in social situations would make it easier for you to live or get along in society? Please answer the choice that best applies to you.

1. Strongly Disagree
2. Disagree
3. Somewhat Disagree
4. Neither Agree nor Disagree
5. Somewhat Agree
6. Agree
7. Strongly Agree

Note: For reasons discussed below, the original item #22 was omitted from the current study. However, this item is still numbered in this supplementary so that the item numbers are consistent with the original checklist for comparison. In the actual survey, this item was not numbered, i.e., the next item after the omitted original item #22 was numbered #22 instead, and the following items were numbered accordingly (i.e., original item number minus one). Therefore, the actual distributed survey had a total of 30 numbered items, as opposed to the 31 numbered items found in this section. The original Japanese items will be appended below the translated text.

1. Avoid social situations where you would stand out.
2. Hold back your thoughts and opinions, or hide aspects of your personality such as hobbies that would be deemed different from the norm*.
3. Suppress atypical behaviors such as hand flapping or fidgeting.
4. Attend social events, even if you would rather not but talk as little as possible*.
5. Try not to stand out (e.g., copy hairstyle, language or interests of others around you) in the group you belong to (e.g., workplace or school).
6. Follow basic social etiquette (e.g., making a smile or providing a backchanneling during conversation)*.
7. Predict, plan out, and rehearse conversations before they happen.
8. Mimic expressions, gestures, facial expressions, tone of voice picked up from other people and/or characters in TV and other media*.
9. Make appropriate eye contact, even if you do not understand the feelings of others when you look into their eyes, or even if you feel aversive. Or, look at close to their eyes such as the bridge of their nose *.
10. Refer to learned conversation patterns, when having conversations (e.g., bring up “safe” topics such as the weather, or use topics that have worked well with others in the past).
11. Steer the conversation to topics you are equipped to talk about (e.g., make the topic something you are good at talking about, such as your interests. Or, focus the conversation on your interaction partner to draw attention away from yourself).
12. When attending social events (e.g., going out to lunch/dinner, hanging out together, going to a drinking party), go with someone who can help you if you say something that is perceived as rude or impolite*.

13. Create an environment in which you do not have to think about too many things at once and can have conversations more easily, or create an environment in which you are less likely to fail (e.g., "flit" between different conversation groups, engage in 1:1 conversations so there are fewer social signals to read, use structured social gatherings such as games where things to do or conversation flow are predetermined)*.
14. Even if it is difficult to imagine the mental state of others, repeat and rephrase what your interaction partner says to give the impression of being a ‘good listener’*.
15. To guide the conversation, use something that can be found in the place (e.g., dog, children, interesting object) as a conversation topic.
16. Play a character of fake self that is inconsistent with the ‘real you’ (e.g., acting confident when you are not, telling fabricated stories, pretending to be sociable when you are nervous being with others).
17. Use learned non-verbal behaviors such as facial expression, body language, or direction of gaze, to infer thoughts or feelings of others (e.g., inferring that when someone yawns during the conversation, they are bored)*.
18. Use learned verbal behaviors, such as tone of voice or context of speech, to infer the thoughts or feelings of others (e.g., inferring that someone who is talking about a funeral with a faint tone is likely to be sad).
19. Infer thoughts or feelings of others based on their past behavior (e.g., if someone invites you out or to a drinking party again, infer that they like you)*.
20. Substitute others with yourself or a TV character to infer their thoughts and feelings*.
21. After social interactions such as a conversation, analyze the situation and think about what you would do the next time the same situation occurs*.
22. This item was not included in the current study*.
23. Infer thoughts and feelings of others accurately by having others repeat what they have said in a different way, learning about them from others, or using more time to think deeply*.
24. Be mindful of your own biases about how you tend to interpret thoughts and feelings of others, and try to understand others accurately (e.g., since I tend to perceive blank facial expressions of others as angry, I try to think that “they are just keeping straight face, even though I perceive them to be angry”).
25. Apply learned psychological theory such as personality traits, to help infer the thoughts and

feelings of others.

26. Navigate conversations by playing to your strengths such as humor or wit.
27. Go out of your way to be helpful to others, so that your social differences might be forgiven.
28. Seek relationships with others who are more accepting of your social differences (e.g., neurodivergent others).
29. Work or study in an environment where your social differences are accommodated (e.g., companies promoting employment for disabled people, workplaces or schools that value the skills you are good at)*.
30. Seek relationships with others who are foreign or live in a foreign country so that your social differences are attributed to the cultural differences.
31. Disclose your difficulties or diagnosis to others around you so that they can create a more comfortable environment for you to be in or to work or study.

Modifications made by the authors:

The authors edited the original Compensation Checklist items to (1) make them easier for participants to understand by shortening them and/or providing examples, (2) focus on concrete behaviors and reduce ambiguity, and (3) adapt them to Japanese cultural contexts (the change in wording has been reflected as much as possible in this supplement while respecting English fluency). The name of each strategy listed in the original Compensation Checklist was not presented to the participants for the sake of brevity.

Notable Changes*:

2, 4, 7, 8, 20, 23: Shortened and simplified for the sake of brevity and ease of understanding.

6: The original example of “look towards other people” was omitted and to provide backchanneling was added instead due to cultural differences. “Manners” has also been omitted due to ambiguity.

9: The original example of “stand at a 90° angle to interaction partner” was omitted due to cultural differences.

10: Original examples of “ask others set questions”, “small talk”, and “turn-take in conversation” were omitted due to ambiguity. “Laugh at joke cues” was omitted due to cultural differences. Instead, “bring up ‘safe’ topics such as weather” and “use topics which worked well with others in the past” were added.

12: Examples of social events have been added for the ease of readers to understand. The expression “social crutch” and the examples ("introduce you, fill in or disguise your social mistakes, explain social nuances to you”) were instead changed to “an individual who can help you if you say something perceived as rude or impolite” due to the cultural difference.

13: Added example for “structured socializing or ‘organized fun” for ease of understanding.

14: Removed the term ‘advisor’ due to cultural differences.

17: The original example of “when someone looks at the ground or rolls their eyes” was replaced with “yawns during the conversation” due to cultural differences.

19: Added examples for “social events” for ease of understanding.

21: Since the first part (“Predict likelihood of what someone is thinking/feeling based on logic, the context or experience of how that person has previously behaved”) was ambiguous, only the latter part was used as a base for this item.

22: This item was omitted in the current study since it was a collection of multiple behaviors ranging from broader concepts and not a single strategy or set of strategies under one concept.

29: The example of “companies promoting employment for disabled people” was added for ease of understanding for Japanese participants, as this is systematized in the Japanese employment system. The term “disabled” here refers to all sorts of conditions related to employment, such as physical, internal, or mental conditions, including neurodevelopmental conditions. The latter examples ("academia, skill-based job”) were generalized since there is no guarantee or consensus that these work environments are more accepting of autistic people.

Subscales (according to the original item numbers, as shown in this supplementary):

Masking: 1-6

Shallow Compensation: 7-16

Deep Compensation: 17-25

Accommodation: 26-31

質問１：

あなたは普段、社会的な場面で以下のような戦略を用いますか。はいかいいえで答えて下さい。

1. はい
2. いいえ

質問２（質問１ではいと答えた場合のみ表示する）：

社会的な場面で以下のような戦略を使うことは、あなたにとってどのくらいとって疲れる、あるいは大変であると感じますか。一番良く当てはまるものを答えて下さい。

1. まったくそう思わない
2. そう思わない
3. あまりそう思わない
4. どちらでもない
5. ややそう思う
6. そう思う
7. 非常にそう思う

質問３（質問１ではいと答えた場合のみ表示する）：

社会的な場面で、以下のような戦略を使うことで、あなたはどのくらい社会の中で行きやすくなったり、うまくやっていけるようになったりすると感じますか。一番良く当てはまるものを答えて下さい。

1. まったくそう思わない
2. そう思わない
3. あまりそう思わない
4. どちらでもない
5. ややそう思う
6. そう思う
7. 非常にそう思う

項目：

1. 周りから目立つような状況をなるべく避ける。
2. 自分の考えや意見を言うのを控えたり、人とは変わった趣味などは言わないようにしたりする。
3. 手をひらひらさせたくなったり、ソワソワしたりしているときでも我慢する。
4. いやいやながらも社会的なイベントや会話に参加するが、発言はできるだけしない。
5. 自分が属する集団（例：職場や学校）の人たちから浮かないようにする（例：髪型、話し方、興味の対象などを周りに合わせてふるまう）。
6. 基本的な社会的マナーに基づいて振る舞う（例：会話をするときに、笑顔を作ったり相槌を打ったりするなど）。
7. 会話をする前に、会話の流れや場面を予測したり、何を話すかをあらかじめ想定したりする。
8. 他の人やテレビなどのキャラクターが使う、言葉遣い、身振り手振り、表情、声の調子を真似る。
9. 目を見ても相手の感情がわからない、あるいは人の目を見るのが嫌であると感じるが、それでも相手の目を見て話す。あるいは、鼻先など目の近くを見て話す。
10. 会話をするときに、いままでに学んだ会話のパターンを参考にする（例：天気などの誰とでも話せる無難な話題や、以前他の人と話して盛り上がった話題を挙げる）。
11. 自分が得意な会話の流れを作る（例：自分が興味を持っていることなど、話すのが得意なことを話題にする。あるいは、話題を相手に関することに集中させて、自分自身を話題にすることを避ける）。
12. 社会的なつながり（例：ご飯に行く、遊びに行く、飲み会に行く）に参加する場合、自分が失礼なことを言ってしまったときに助けてくれるような人と一緒に行く。
13. 自分があれこれ考える必要がなく楽に話せたり、失敗しづらい環境を作る（例：異なる会話のグループを行ったり来たりする。相手の顔色を伺う必要が少なくなるように１対１で話す。ゲームややることがはっきり決まっている集まりなど、やることや話の進め方が決まっているかかわりを活用する）。
14. 相手の心をうまく想像できない場合でも「良い聞き手である」という印象を与えられるように、相手の言ったことを繰り返したり、別の表現で言い直したりする。
15. 会話の流れを作るために、その場にあったり、いたりするもの（例：犬、子ども、面白いもの）を話の種にする。

16. 本当の自分とは違う、作った自分を演じる（例：本当は自信がないのに自信がある振りをする。嘘の話をする。他者と一緒にいるのが苦手でも友達付き合いが好きなふりをする）。
17. これまでに学んだ、表情、ボディランゲージ、視線などの非言語的振る舞いをもとに他者の考えや感情を推測する（例：話している最中にあくびをしている人がいたら、話に退屈しているのだろうと推測する）。
18. これまでに学んだ、声の調子や話の文脈などの言語的な振る舞いをもとに、他者の考えや感情を推測する（例：葬式について弱々しい声で話している人は、悲しいのだろうと推測する）。
19. 相手の過去の振る舞いをもとに、相手の考えや感情を推測する（例：遊びに行こう、飲みに行こうと何度も誘われたら、その人は自分のことが好きだと推測する）。
20. 相手のことを、自分やテレビのキャラクターなどに置き換えることで、その人の考えや感情を推測する。
21. 会話などのやりとりをした後、そのときの状況を自分なりに分析して、次に同じような場面が起きたときはどうするのがよいか考える。
22. この項目は本研究では用いなかった。
23. 相手が言ったことを別の言い方で繰り返してもらったり、その人に関することについて他の人から意見をもらったり、時間をかけてじっくり考えたりすることで、他の人の考えや感情を精密に推測する。
24. 相手の考えや感情を自分がどう捉えやすいかという、自分の癖に気をつけることで、正しく相手を理解しようとする（例：自分は普通の表情を怒っていると感じやすいので、相手の表情を怒っていると感じたときは、「怒っているようにみえるけど、これは普通の表情だ」と考えるようにする）
25. パーソナリティ特性など、心理学の理論を活用することで相手の考えや感情を推測しようとする。
26. ユーモアや知性といった、自分の強みを生かして会話を進める。
27. 自分から人の役に立つようにすることで、自分の「変わっているところ」が許されるようにする。
28. 自分の社会的なあり方を受け入れてくれるような人たち（例：他の発達障がいを持つ人たち）と付き合うようにする。
29. 自分の社会的あり方を受け入れてくれるような場所（例：障がい者雇用を促進してい

る職場、自分が得意なスキルを重視している職場・学校）で働く・勉強する。

30. 外国人と付き合ったり、外国に住んだりすることで、自分の社会的あり方は文化の違いだと思ってもらうようにする。
31. 自分が過ごしやすかったり、仕事や勉強をしやすかったりする環境づくりのために、診断のことや困っていることを周りの人に伝える。

Figure S1

Flowchart of Participant Recruitment and Screening Procedure

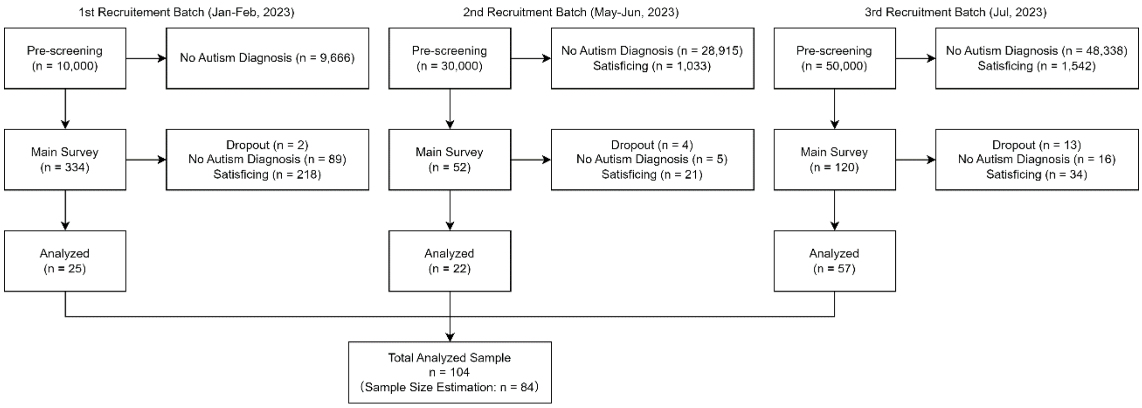

Figure S2

Survey Structure

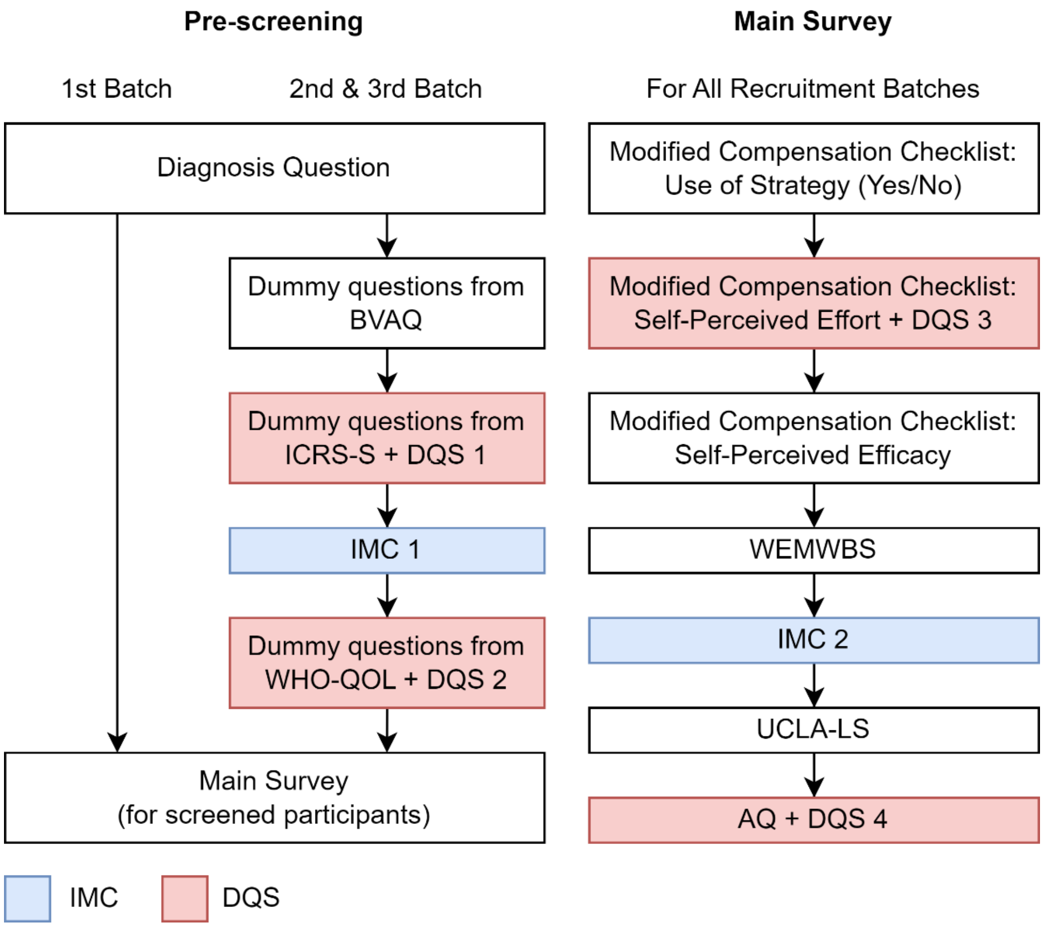

Note: For the pre-screening, participants recruited in the first batch completed only the diagnosis question. Those recruited in the second and third batches completed additional screening questions, including 1 IMC and 2 DQS questions. The initial screening questions were extracted from different questionnaires and were used purely as dummy questions to detect participants who satisficed and were therefore not used in the analysis. Only those who passed the pre-screening were given the main survey. The main survey was identical for participants recruited in all batches. To detect satisficing throughout, as opposed to at one point in the survey, 2 DQS questions sandwiched 1 IMC question in order in both the screening and the main survey, and they were placed apart. For each DQS and IMC items, refer to Methods S4 and S5, respectively.

IMC = Instructional Manipulation Check; DQS = Directed Questions Scale; BVAQ = Bermond-Vorst Alexithymia Questionnaire; ICRS-S = Integrating Conflict Resolution Skills Scale; WHO-QOL = The World Health Organization Quality of Life; WEMWBS = Warwick-Edinburgh Mental Wellbeing Scale; UCLA-LS = UCLA Loneliness Scale Version 3; AQ = Autism-Spectrum Quotient.

Figure S3

Distribution of self-perceived effort and efficacy of using social strategies

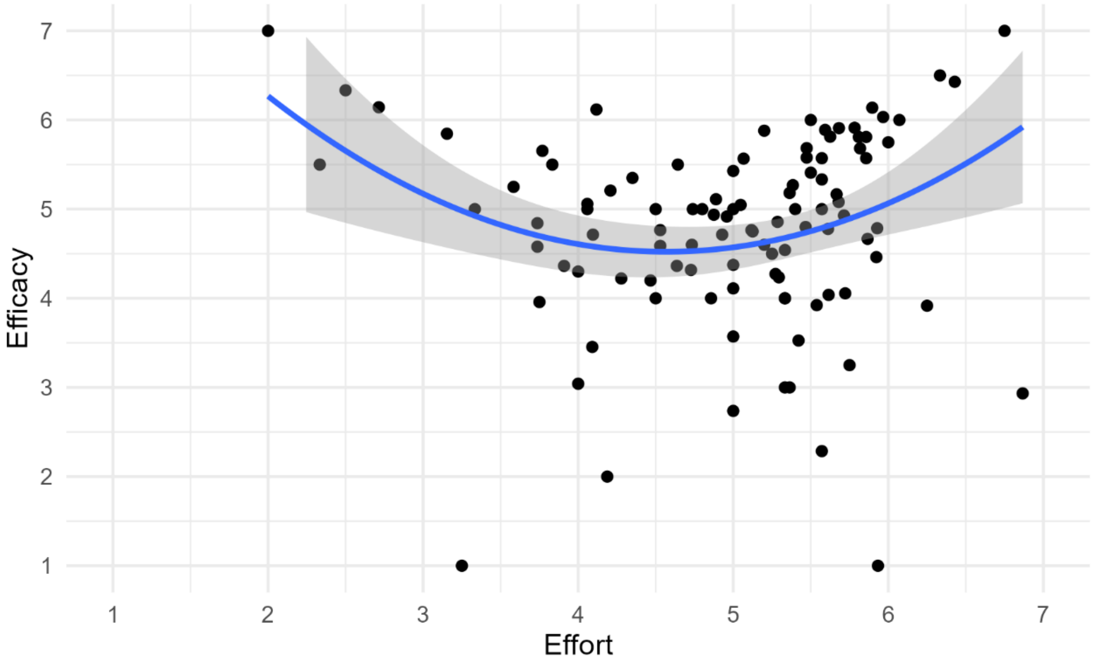

Note: A linear regression with quadratic terms was conducted to examine the relationship between overall self-perceived effort and efficacy (efficacy ~ effort + [effort ^ 2] + intercept). The model was significant (p = 0.010), although the explanatory power was weak (R^2 = 0.07). The effect of effort was significant (β = 0.18, p = 0.003), and the effect of effort squared was also significant (β = 0.21, p = 0.002). This quadratic distribution between self-perceived effort and efficacy may explain the non-significant effect of the interaction effect between self-perceived effort and efficacy on autistic well-being in the main regression analysis, as it may reduce the variance of the interaction term.

Figure S4

Visualization of linear regression of association between WEMWBS and self-perceived effort and efficacy of social strategies used in each MCC subscale

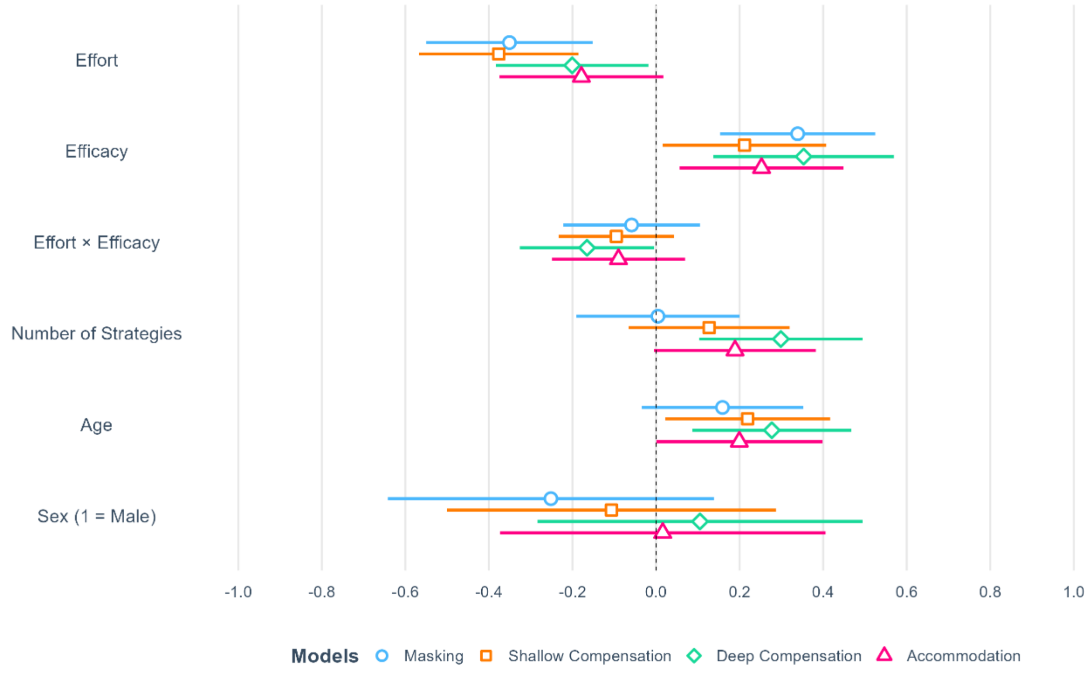

Note: Bars represent 95% confidence intervals. Participants who do not use any strategies within each subscale are excluded from the analysis. Masking (n = 96), shallow compensation (n = 99), deep compensation (n = 96), accommodation (n = 94).

Figure S5

Visualization of linear regression of association between UCLA and self-perceived effort and efficacy of social strategies used in each MCC subscale

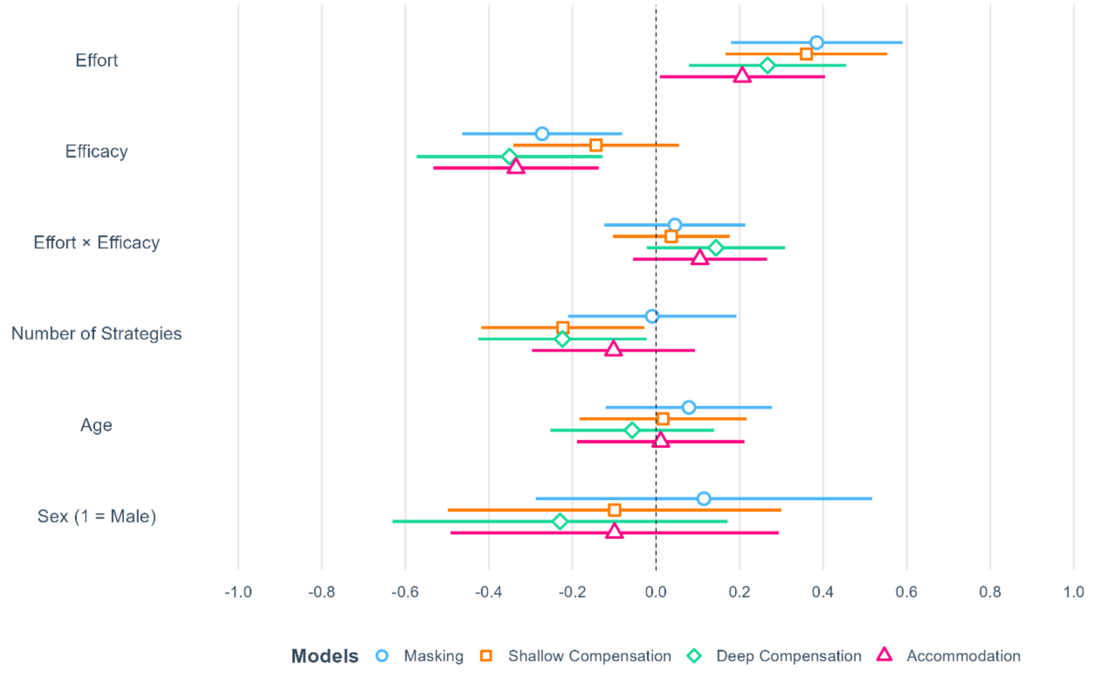

Note: Bars represent 95% confidence intervals. Participants who do not use any strategies within each subscale are excluded from the analysis. Masking (n = 96), shallow compensation (n = 99), deep compensation (n = 96), accommodation (n = 94).

Figure S6

Interaction effect of self-perceived effort and efficacy using deep compensation strategies in WEMWBS

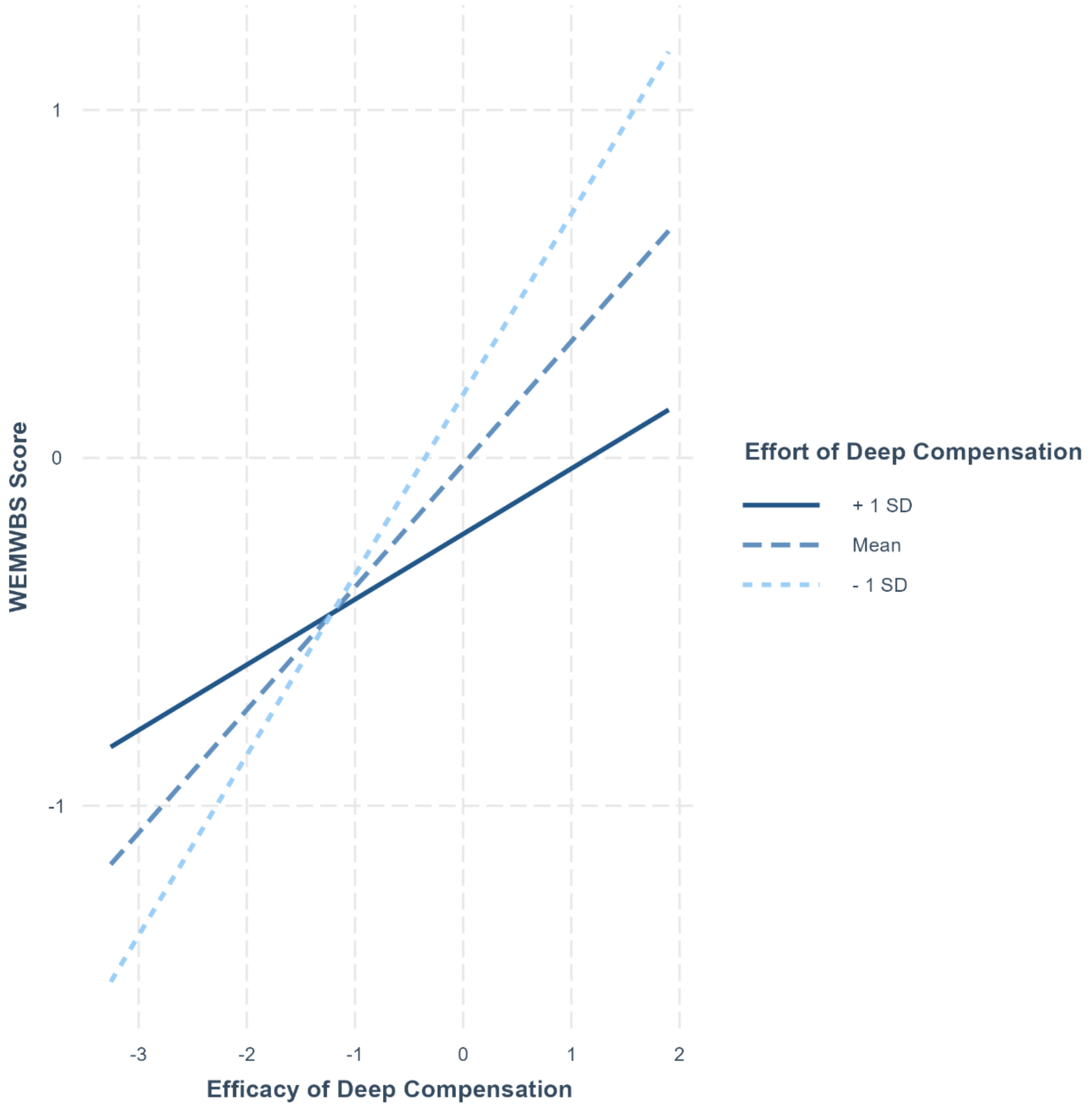

Note: N = 96. WEMWBS = Warwick-Edinburgh Mental Wellbeing Scale.

Table S1

Regression of association between WEMWBS and self-perceived effort and efficacy, excluding participants with IDD and/or SLD.

Variables	Excluding IDD (n = 98)				Excluding SLD (n = 99)				Excluding IDD & SLD (n = 96)			
	β	95% CI		p	β	95% CI		P	β	95% CI		p
		LL	*UL*			*LL*	*UL*			*LL*	*UL*	
Effort	-.37	-.56	-.18	<.001	-.43	-.61	-.25	<.001	-.40	-.59	-.22	<.001
Efficacy	.35	.18	.53	<.001	.30	.13	.47	<.001	.34	.16	.51	<.001
Effort × Efficacy	.00	-.12	.13	.948	-.03	-.15	.10	.677	-.01	-.13	.12	.912
Sex [a]	.01	-.36	.37	.978	.08	-.27	.44	.647	.03	-.34	.39	.883
Age	.18	.00	.36	.051	.14	-.04	.31	.119	.17	.00	.35	.055
Number of Strategies	.18	.00	.37	.056	.22	.04	.39	.018	.16	-.03	.34	.095
Adjusted R^2	.288				.312				.304			
p	<.001				<.001				<.001			

Note: N = 104. IDD = Intellectual Developmental Disorder; SLD = Specific Learning Disorder; CI = confidence interval; *LL* = lower limit; *UL* = upper limit.

[a] 0 = female, 1 = male.

Table S2

Regression of association between UCLA-LS and self-perceived effort and efficacy, excluding participants with IDD and/or SLD.

Variables	Excluding IDD (n = 98)				Excluding SLD (n = 99)				Excluding IDD & SLD (n = 96)			
	β	95% CI		p	β	95% CI		P	β	95% CI		p
		LL	*UL*			*LL*	*UL*			*LL*	*UL*	
Effort	.44	.25	.63	<.001	.46	.28	.64	<.001	.45	.26	.64	<.001
Efficacy	-.29	-.47	-.11	.002	-.26	-.43	-.09	.003	-.28	-.46	-.11	.002
Effort × Efficacy	.04	-.09	.17	.527	.05	-.07	.18	.396	.04	-.08	.17	.510
Sex [a]	-.07	-.44	.30	.711	-.05	-.41	.31	.788	-.04	-.41	.33	.824
Age	.04	-.14	.22	.689	.06	-.11	.24	.463	.05	-.13	.23	.565
Number of Strategies	-.21	-.40	-.02	.030	-.22	-.40	-.04	.015	-.20	-.39	-.01	.038
Adjusted R^2	.279				.305				.286			
p	<.001				<.001				<.001			

Note: N = 104. IDD = Intellectual Developmental Disorder; SLD = Specific Learning Disorder; CI = confidence interval; *LL* = lower limit; *UL* = upper limit.

[a] 0 = female, 1 = male.

Table S3

Regression of association between WEMWBS and self-perceived effort and efficacy of used social strategies in each CC subscale.

Variables	Masking (n = 96)				Shallow Compensation (n = 99)				Deep Compensation (n = 96)				Accommodation (n = 94)			
	β	95% CI		p	β	95% CI		p	β	95% CI		p	β	95% CI		p
		LL	*UL*			*LL*	*UL*			*LL*	*UL*			*LL*	*UL*	
Effort	-.35	-.55	-.15	<.001	-.38	-.57	-.19	<.001	-.20	-.38	-.02	.031	-.18	-.37	.02	.073
Efficacy	.34	.15	.52	<.001	.21	.02	.41	.034	.35	.14	.57	.002	.25	.06	.45	.012
Effort × Efficacy	-.06	-.22	.11	.479	-.10	-.23	.04	.175	-.17	-.33	.00	.044	-.09	-.25	.07	.265
Sex [a]	-.25	-.64	.14	.204	-.11	-.50	.29	.592	.11	-.28	.49	.593	.20	-.37	.41	.935
Age	.16	-.03	.35	.105	.22	.02	.42	.030	.28	.09	.47	.005	.20	.00	.40	.049
Number of Strategies	.00	-.19	.20	.963	.13	-.07	.32	.194	.30	.10	.49	.003	.19	.00	.38	.055
Adjusted R^2	.196				.161				.244				.166			
p	<.001				.001				<.001				.001			

Note: CI = confidence interval; *LL* = lower limit; *UL* = upper limit.

[a] 0 = female, 1 = male.

Table S4

Regression of association between UCLA-LS and self-perceived effort and efficacy of used social strategies in each CC subscale.

Variables	Masking (*n* = 96)				Shallow Compensation (*n* = 99)				Deep Compensation (*n* = 96)				Accommodation (*n* = 94)			
	β	95% CI		*p*	*β*	95% CI		*p*	*β*	95% CI		*p*	*β*	95% CI		*p*
		LL	*UL*			*LL*	*UL*			*LL*	*UL*			*LL*	*UL*	
Effort	.38	.18	.59	<.001	.36	.17	.55	<.001	.27	.08	.45	.006	.21	.01	.40	.041
Efficacy	-.27	-.46	-.08	.006	-.14	-.34	.05	.154	-.35	-.57	-.13	.002	-.34	-.53	-.14	.001
Effort × Efficacy	.05	-.12	.21	.596	.04	-.10	.18	.606	.14	-.02	.31	.089	.11	-.06	.27	.197
Sex [a]	.11	-.29	.52	.573	-.10	-.50	.30	.623	-.23	-.63	.17	.258	-.10	-.49	.29	.617
Age	.08	-.12	.28	.434	.02	-.18	.22	.868	-.06	-.25	.14	.564	.01	-.19	.21	.911
Number of Strategies	-.01	-.21	.19	.929	-.22	-.42	-.03	.026	-.22	-.43	-.02	.030	-.10	-.30	.09	.303
Adjusted R^2	.145				.138				.197				.152			
p	.003				.003				<.001				.002			

Note: CI = confidence interval; *LL* = lower limit; *UL* = upper limit.

[a] 0 = female, 1 = male.
